# Supplementary material for: Factors related to subjective well-being among community-dwelling older adults living alone: A stratified analysis by sex and marital status from the JAGES
Source: PLoS One. 2023 Aug 22;18(8):e0289571. doi: 10.1371/journal.pone.0289571 (PMC10443856; doi:10.1371/journal.pone.0289571)
Supplement: Attachment — Submitted filename: Response to Reviewers.docx [file pone.0289571.s001.docx]

Response to Reviewers

**To Reviewer #1**

***Thank you for the thoughtful and constructive feedback you provided regarding our manuscript, Factors Related to Subjective Well-being Among Community-Dwelling Older Adults Living Alone: A Stratified Analysis by Sex and Marital Status from the JAGES (PONE-D-23-04435).***

1. I am really confused about the objective and hypothesis of the present study.

⇒***The number of older adults living alone, which is expected to increase in Japan and other developed countries, is likely to be diverse with regard to sex and marital status. Therefore, focusing on the four population categories of older adults living alone based on sex and marital status (married men, unmarried men, married women and unmarried women) —rather than on the overall group of older adults living alone—while also focusing on not only negative health outcomes but also positive health outcomes such as SWB is necessary. Moreover, studying representative populations in longitudinal studies from which causal relationships between SWB over time and their association can be inferred is critical for developing evidence-based health policy that is responsive to new populations and diversity. Therefore, in this study, by using a longitudinal study of a representative population (the Japan Agency for Gerontological Evaluation Study), our aim was to determine changes in SWB over time and their association with independent functioning factors and interpersonal factors by sex and marital status (married men, unmarried men, married women and unmarried women) among older adults living alone in the community. Independent functioning factors are critical for performing daily living tasks without help [21, 22]. The achievement of functional independence ensures that older adults can participate fully in meaningful and purposeful life events—a requirement for SWB. Interpersonal factors are individual elements or a group of factors that positively or negatively influence the quality of relationships [21, 23] and are integral to creating and maintaining meaningful personal relationships that ensure SWB among older adults in the community. Our hypotheses were as follows:***

***Hypothesis 1: SWB is more likely to transition to a high level in women rather than men and in married versus unmarried individuals among community-dwelling older adults living alone.***

***Hypothesis 2: Independent functioning factors and interpersonal factors are significantly associated with SWB in older adults living alone and differ by sex and marital status among community-dwelling older adults living alone. (L95～)***

2. I think authors have done the polled data analysis...If yes? why they did not adopted other longitudinal data analysis techniques?

3. The data is so rich; therefore using a pooled data analysis which is largely appropriate for cross-sectional studies is a matter of concern for me.

***⇒This study is not a polled data analysis but a longitudinal data analysis (⇒the statistical method).***

**To Reviewer #2**

***Thank you for the thoughtful and constructive feedback you provided regarding our manuscript, Factors Related to Subjective Well-being Among Community-Dwelling Older Adults Living Alone: A Stratified Analysis by Sex and Marital Status from the JAGES (PONE-D-23-04435).***

This study examined factors related to subjective well-being (SWB) among Japanese community-dwelling elderly stratified by sex and marital status. This manuscript may contribute to this area of research. Further attention to the issues presented below would strengthen the manuscript.

Major points

#1Regarding participant groups.

In this study, only older adults who live alone were included in the analysis, and they were divided into groups by gender and marital status to compare their SWB.

a) It would be better to state in the text body about the reason why the analysis was conducted only for those living alone in this study. If the authors are interested in the SWB states of older adults who live alone, it would be better to have a comparison group (i.e., older individuals who live with their families) to better clarify the nature of older adults living alone. b)For those who are married but living alone, it would be helpful to specify their specific situation in the method (e.g., married, but their husband institutionalized?). How many of these are included in the data set? Are there any problems in analyzing these as the same group as those who have divorced from spouses or have been bereaved of his/her spouse?

***⇒Thank you for your comments. 　a) We agree with you that it would be better to have a comparison group (i.e., older individuals who live with their families) to better clarify the nature of older adults living alone. However, while it is already clear that*** ***older adults living alone are risk factors for a various health outcome, including SWB, compared to older adults living with family members, it is not clear whether SWB and related factors differ by sex and marital status among older adults living alone. Therefore, in this study, we analyzed a sample of older adults living alone, stratified by sex and marital status (married men, non-married men, married women, and non-married women).　b) Those who are married but living alone situation assumes that the spouse is an institutionalized resident, as you pointed out. Since the main purpose of this survey is to compare men and women, married and unmarried, those who were divorced, bereaved, or separated (institutionalized) were included in the married group. We would like to research on comparison of divorced, bereaved, or separated (institutionalized) in the married group as you recommended*** ***in the next step.*** ***Thank you for the thoughtful feedback.***

***Additional information: The married men group consisted of 1,803 men, among whom 63 (3.5%) were currently married, 1,005 (55.7%) were widowed, and 735 (40.8%) were divorced. The married women group consisted of 1,803 women, among whom 63 (3.5%) were married, 1005 (55.7%) were widowed, and 735 (40.8%) were divorced. In addition, individuals in the “married with a spouse” group were assumed to be those whose spouses lived alone because they were moved to an institution or for other reasons.　(L.152～)***

#2

Regarding outcomes.

The authors should specify in the statistical analyses section how the outcomes (binary values) of the longitudinal analyses were defined and analyzed. Also, since it appears that the baseline values were controlled for in the multivariate analysis (Table 5), the statistical adjustments should be clearly stated in the text. In addition, the proportion of events occurring in the outcome should be noted in the results.

***⇒Thank you for your important comments. We have added a note about a detailed description of the analytical method in statistical analyses section, as follows: The logistic regression analysis was conducted with the dependent variables as “1” for high level SWB in 2019 and “0” for low level SWB in 2019. The analysis was conducted with sex and SWB level at baseline as adjustment variable (L.229～).***

***This study did not evaluate the occurrence of SWB-related events (e.g., death of a friend). This is the next issue as you commented.***

#3

Regarding multivariate analysis.

It should be clearly stated whether all the independent variables were entered simultaneously or not in multivariate analysis. In particular, interpersonal relationship factors include variables that have a high correlation with each other, but are there any problems in such analysis? Is there any possibility of over-adjustment?

Similarly, if the subscales of the TMIG-IC were simultaneously entered into the model, would this pose any problems? It is theoretically unlikely that the total score and the subscale score would be entered at the same time, I believe. The authors' opinion should be stated on this point.

***⇒As you indicated, we did not perform a multivariate analysis because of multicollinearity. Univariate logistic regression analyses were performed for each independent variable, and in each univariate logistic regression analysis, gender and SWB level at baseline were entered and adjusted. Similarly, univariate logistic regression analysis was also performed for the total score and subscales, taking into account*** ***multicollinearity for the TMIG-IC.***

Minor points

#4

In Table 5, TMIG-IC subscale names should be used from the official names provided by the previous study (Koyano et al. 1991).

***⇒We are sorry. The Tokyo Metropolitan Institute of Gerontology Index of Competence (TMIG-IC). [22]. (L.181～)***

#5

Typos?

Notes in Table5. SWB H and group --> SWB H and L group

***⇒We are sorry. Notes in Table5 has been revised.***

**To Reviewer #3**

***Thank you for the thoughtful and constructive feedback you provided regarding our manuscript, Factors Related to Subjective Well-being Among Community-Dwelling Older Adults Living Alone: A Stratified Analysis by Sex and Marital Status from the JAGES (PONE-D-23-04435).***

1. In the Abstract, what is the meaning of "SWB" ? Pleased add the information at this word firstly appeared.

***⇒We are sorry and thank you for your comment. We added the information of subjective well-being (SWB) in the Abstract.***

2. In the table 1, add the collected time of variables.

***⇒******Thank you for your suggestion. We added the collected time of variables in table 1.***

3. “How happy are you currently? Please provide a score on a 10-point scale from 0 (very unhappy) to 10 (very happy).” Is this question suitable for assessing the SWB? Added reference.

***⇒Thank you for your suggestion. The SWB 10-point scale has been used in JAGES. We have added a reference regarding SWB in JAGES [21].***

4. How to assess the transitions of marital status from 2016 to 2019? And presented the association between the transition of SWB and transitions of marital status?

***⇒******Thank you for your comment. We did not assessed the transitions in marital status from 2016 to 2019 since we exclude subject whose marital status has changed.***

5.Why used logistic regression inseaded of COX?

***⇒******Thank you for your comment. We used logistic regression since Cox proportional hazards regression assumes proportionality, but logistic regression is not subject to this restriction.***
