# Supplementary material for: Factors related to subjective well-being among community-dwelling older adults living alone: A stratified analysis by sex and marital status from the JAGES
Source: PLoS One. 2023 Aug 22;18(8):e0289571. doi: 10.1371/journal.pone.0289571 (PMC10443856; doi:10.1371/journal.pone.0289571)
Supplement: Attachment — Submitted filename: Response to Reviewers.docx [file pone.0289571.s002.docx]

Response to Reviewers

***To Reviewer #2:***

***We sincerely*** ***thank you again for the thoughtful and constructive feedback you provided regarding our manuscript, PONE-D-23-04435R1：Factors Related to Subjective Well-being Among Community-Dwelling Older Adults Living Alone: A Stratified Analysis by Sex and Marital Status from the JAGES.***

Regarding #1.

I understand the rational for participant classification regarding marital status. Thank you so much. By the way, in the "additional information" (L152-), the number of persons (percentages) for each classes in married men and women were identical. Please confirm it.

⇒***We apologize for our typo and thank you for your careful peer review.*** ***We have made the following corrections：***

***Additional information: The married men group consisted of 1,803 men, among whom 63 (3.5%) were currently married, 1,005 (55.7%) were widowed, and 735 (40.8%) were divorced.*** ***The married women group consisted of 5,372 women, among whom 54 (1.0%) were married, 4,319 (80.4%) were widowed, and 999 (18.6%) were divorced. In addition, individuals in the “married with a spouse” group were assumed to be those whose spouses lived alone because they were moved to an institution or for other reasons.　(L.152～)***

Regarding #3.

Thank you for explaining regarding "whether all the independent variables were entered simultaneously or not in multivariate analysis". Since this is very important information for interpreting findings, the authors should clearly state the information in the text body (in the statistical analyses section), I believe.

⇒***Thank you for your important comments. We have added a note about a detailed description of the analytical method in statistical analyses section, as follows: Multivariate analyses were not performed for each independent variable, because of multicollinearity. Univariate logistic regression analyses were performed for each independent variable, and in each univariate logistic regression analysis, gender and SWB level at baseline were entered and adjusted to determine whether the 3-year change in SWB was associated with independent functional factors and interpersonal relationship factors, adjusting for age and SWB level at baseline. (L.231～)***

Reviewer #3: This study analyzed the changes in subjective well-being over time and the factors associated with this change. The finding will be useful for policy-making to promote subjective well-being. In the current manuscript, author has addressed my all comments.

***⇒Thank you again for the constructive feedback.***
